# Comparative Study of Protective Effects of Salbutamol and Beclomethasone against Insulin Induced Airway Hyper-reactivity on Isolated Tracheal Smooth Muscle of Guinea Pig

**Published:** 2015

**Authors:** Mahjabeen Sharif, Bushra Tayyaba Khan, Salman Bakhtiar, Mohammad Asim Anwar

**Affiliations:** a *Department of Pharmacology and Therapeutics, National University of Sciences and Technology (NUST) Islamabad,Army Medical College Rawalpindi, Pakistan.*; b *Department of Pharmacology and Therapeutics, Army Medical College Rawalpindi,National University of Sciences and Technology (NUST) Islamabad,Pakistan.*; c *Department of Pharmacology and Therapeutics Army Medical College Rawalpindi,National University of Sciences and Technology (NUST) Islamabad, Pakistan.*; d *Consultant Physician Pakistan Atomic Energy Commission Hospital (PAEC) Islamabad.*

**Keywords:** Airway hyper-reactivity, Inhaled insulin, Salbutamol, Beclomethasone, Tracheal muscle

## Abstract

Inhalational insulin was withdrawn from the market due to its potential to produce airway hyper-reactivity and bronchoconstriction. So the present study was designed to explore the acute effects of insulin on airway reactivity of guinea pigs and protective effects of salbutamol and beclomethasone against insulin induced airway hyper-responsiveness on isolated tracheal smooth muscle of guinea pig. Effects of varying concentrations of insulin (10^-7^ to 10^-3^ M), insulin pretreated with fixed concentration of salbutamol (10^-7^ M) and beclomethasone (10^-6^ M) were studied on isolated tracheal tissue of guinea pig by constructing cumulative concentration response curves. Changes in tracheal smooth muscle contractions were recorded on four channel oscillograph. The mean ± SEM of maximum amplitudes of contraction with increasing concentrations of insulin, insulin pretreated with fixed concentration of salbutamol and beclomethasone were 35 ± 1.13 mm, 14.55 ± 0.62 mm and 22 ± 1.154 mm respectively. Although salbutamol and beclomethasone both had a profound inhibitory effect on insulin induced airway hyper-reactivity, yet salbutamol is more efficacious than beclomethasone. So we suggest that pretreatment of inhaled insulin with salbutamol may be preferred over beclomethasone in amelioration of its potential respiratory adverse effects such as bronchoconstriction.

## Introduction

Subcutaneous insulin is the mainstay for controlling blood glucose in diabetes. Noninvasive, inhalational insulin is an attractive alternative to parenteral insulin for those patients who defer to initiate subcutaneous insulin (1). Studies reveal that inhalational insulin thrice daily before meals can provide glycemic control comparable to conventional subcutaneous insulin but with improved patient’s satisfaction and compliance (2). Long term studies have also demonstrated a significant reduction in HbA_1c _ with fewer hypoglycemic episodes and less risk for weight gain as compared to regular insulin (3). Unfortunately it was withdrawn from the market due to its respiratory adverse effects such as increased bronchial reactivity, cough, dyspnoea and bronchoconstriction (4). Insulin has long been recognized as pro-inflammatory and pro-contractile hormone (5). The most likely mechanism of inhaled insulin induced bronchoconstriction is that insulin modulates the mast cells degranulation and subsequently increased release of histamine and contractile prostaglandins are responsible for allergic inflammation of airways (6). Some experimental evidences also reveal that it is likely to be vagally mediated and increased release of acetylcholine is responsible for air-way hyper-responsiveness (7). Various therapeutic strategies have been implicated to decrease the airway hyper-reactivity mediated by inhaled insulin. Previous studies demonstrate that salbutamol acts as a physiological antagonist and reverses the bronchoconstriction irrespective of bronchoconstrictor stimuli (8). Experimental and clinical evidences have also shown that beclomethasone prevents the allergen induced bronchial reactivity due to its ability to prevent the release of contractile prostaglandins and histamine from mast cells (9). In several studies beclomethasone has also been found to inhibit vagally mediated contractile response of guinea pig airways (10). Insulin induced isolated tracheal muscle contraction in guinea pig model described in the present study closely resembles the bronchoconstriction induced by pulmonary delivery of inhaled insulin as high concentration of insulin gets deposited in airway smooth muscle compartment in both cases (4). So keeping in view the above mentioned pharmacological effects of salbutamol and beclomethasone, the current experimental study was designed to explore and compare the efficacy of salbutamol and beclomethasone against insulin mediated tracheal tissue contraction of guinea pig *in-vitro*. 

## Experimental

*Material and methods*

The study was conducted in the department of Pharmacology & Therapeutics in collaboration with Centre for Research in Experimental and Applied Medicine (CREAM) Army Medical College, Rawalpindi from December 2011 to July 2012. 

*Animals*

The current study was conducted on the isolated tracheal smooth muscle of 18 guinea pigs of Dunkin Hartley variety weighing 500 to 700 g. They were kept in the animal house of Army Medical College, Rawalpindi for one week at 37 ^o^C under 12 hour normal phase light-dark cycle for acclimatization (11). All the protocols described in this study were approved by Ethics committee of Centre for Research in Experimental and Applied Medicine (CREAM) Army Medical College, Rawalpindi.

*Drugs*

 Salbutamol sulphate (5 mg/mL) and beclomethasone dipropionate (0.4 mg/mL) were purchased from Glaxosmithkline. Regular human insulin (100 IU/mL) was obtained from Lilly Pharma.

*Experimental setup*

18 guinea pigs were randomly divided into three groups. They were killed by cervical dislocation (11). The trachea was dissected out and tracheal chain was prepared with smooth muscle in the centre and cartilaginous portions on both sides. One end of the tracheal strip was attached to the hook of oxygen tube of tissue bath containing oxygenated krebs-Henseleit solution at 37 ^o^C, while the other end was connected to the Transducer (Harvard Model No 72-4494). Four channel oscillograph Harvard Model No 50-9307 (England) was used for recording the tracheal muscle contraction (12). 

*Experimental groups*

In group 1, cumulative concentraton response curves of insulin were obtained by using the concentrations ranging from of 10^-7^ to 10^-3^M (4). When the plateau was achieved with first dose (10^-7^M) of insulin, then the next dose (10^-6^ M) was added without washing the previous dose. Changes in tracheal smooth muscle contractions were recorded on oscillograph. When maximal insulin induced contraction was obtained with 10^-3^M concentration of insulin, the tracheal strip was washed three to four times. This group served as control group. In group 2, salbutamol was added to the organ bath in a concentration of 10^-6^ M (13). After 15 minutes, the successive doses of insulin ranging from 10^-7^ to 10^-3 ^M were added into the organ bath in the presence of salbutamol. Cumulative concentration response curves pretreated with salbutamol were constructed. In group 3, cumulative concentration response curves of insulin pretreated with fixed concentration (10^-6^ M) of beclomethasone (14) were constructed. 

*Statistical analysis*

The results were expressed as Means + Standard Error of Means and statistically significant differences were assessed by one way ANOVA followed by Post Hoc Tuckey Test using SPSS version 16. The differences between the observations were considered as significant if p-value was less than 0.05.

## Results

**Figure 1:**
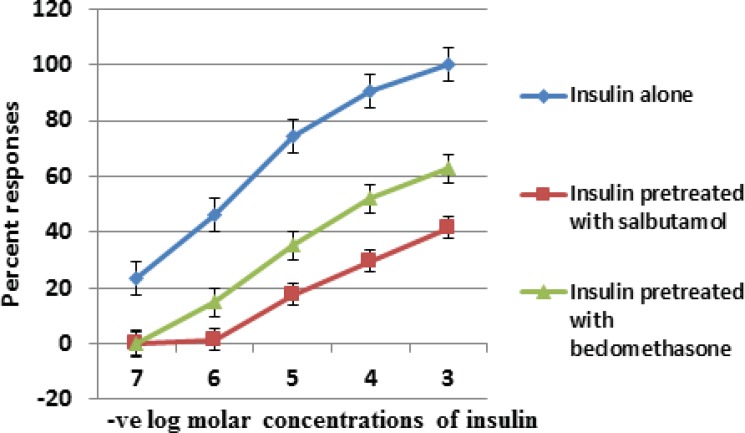
Comparison of semi log concentration response curve of group 1 (insulin control) with group 2 (insulin after pretreatment with salbutamol) and group 3 (insulin after pretreatment with beclomethasone) on isolated tracheal smooth muscle of guinea pig. Results are average of six separate experiments. Data is represented as mean ± standard error of means (SEM).

We studied the acute effects of insulin on isolated tracheal smooth muscles of guinea pig. Insulin induced a dose dependent reversible contraction of tracheal smooth muscle (Figure 1). Changes in tracheal smooth muscle contractions were measured by taking the amplitude of contraction. Maximum amplitude of contraction with 10^-3^ M concentration of insulin was 35 ± 1.13 mm. So insulin directly enhanced the myogenic airway smooth muscle tone. This insulin induced tracheal smooth muscle contraction was significantly reduced in salbutamol and beclomethasone treated groups from 35 ± 1.13 mm (control) to 14.55 ± 0.62 mm and 22 ± 1.154 mm respectively (Table 1). The means of amplitudes of contraction with varying doses of insulin (10^-7^ -10^-3^M) when compared between group 1, 2 and 3, were found to be statistically significant (Table 1).

The percentage responses for all the three groups were also calculated. Our data showed that maximum constrictor response of insulin in the presence of salbutamol and beclomethasone was reduced by 41.57 and 62.86 percent respectively as compared with control group (Table 1). 

**Table 1:** Comparisons of means of amplitudes of contractions and percent responses of isolated tracheal smooth muscle of guinea pig to insulin control (group 1), with insulin pretreated with salbutamol (group 2) and beclomethasone (group 3).

**Concentration of insulin (M)**	**Amplitude of contraction with insulin ** ** (n=6)** **(mean ± S.E.M)** ** (mm) **	**Amplitude of contraction with insulin pretreated with ** **salbutamol (n=6)** ** (mean ± S.E.M)** ** (mm) **	**Amplitude of contraction with insulin pretreated with beclomethasone ** ** (n=6)** ** (mean ± S.E.M)** ** (mm) **	**p-value **	**Percent** **response with insulin**	**Percent response with insulin pretreated with salbutamol **	**Percent response with insulin pretreated with beclomethasone**
10^-7^	8.167 ± 0.87	0 ± 0	0 ± 0	.000*	23.34	0	0
10^-6^	16.16 ± 1.01	0.5 ± 0.34	5.167 ± 0.83	.000*	46.17	1.43	14.77
10^-5^	26.1 ± 1.13	6.17 ± 0.477	12.33 ± 1.08	.000*	74.58	17.62	35.23
10^-4^	31.8 ± 0.832	10.33 ± 0.67	18.17 ± 1.045	.000*	90.86	29.5	51.91
10^-3^	35 ± 1.13	14.55 ± 0.62	22 ± 1.154	.001*	100	41.57	62.86

(*) p-value < 0.05 = Significant

Insulin concentration response curve in the presence of salbutamol was shifted to the right and downwards more than beclomethasone (Figure 1). Although salbutamol and beclomethasone both had a profound inhibitory effect on airway hyper-reactivity induced by insulin yet salbutamol is more efficacious than beclomethasone (Figure 1).

## Discussion

The present study was carried out to explore and compare the protective effects of salbutamol and beclomethasone against insulin induced tracheal tissue contraction. Insulin produced a concentration dependent, reversible contraction of tracheal smooth muscle. These findings were consistent with the results of Schaafsma and his colleagues who also reported the acute contractile effect of insulin due to increased release of contractile prostaglandins on isolated tracheal smooth muscle of guinea pig (4). Our findings are also supported by *in-vivo* studies in which ovalbumin challenged diabetic rats when treated with insulin, the airway inflammation and reactivity was aggravated due to increased release of inflammatory mediators from mast cells (15).

 To ameliorate the airway hyper-reactivity induced by insulin, tracheal smooth muscle was pretreated with salbutamol. Concentration response curve of insulin in the presence of salbutamol was shifted downwards and to the right indicating a profound inhibitory effect on insulin mediated airway smooth muscle contraction. These potential protective effects of salbutamol against insulin mediated tracheal contraction is presumably through its ability to prevent the release of inflammatory mediators from tracheal strip of guinea pig (13). The beneficial effect may also be ascribed due to by its ability to reduce the cholinergic neurotransmission in airway smooth muscles by an action on presynaptic heterogenous β_2_ receptors to inhibit Acetylcholine release (13). Our findings are in agreement with the clinical observations in which inhalation of albuterol 30 minutes before the administration of inhaled insulin increased the absorption of inhaled insulin due to reduction of bronchoconstriction induced by inhaled insulin in asthmatic patients (16).

Beclomethasone also inhibited the insulin induced tracheal smooth muscle contraction by shifting the concentration response curve to the right and downwards. The concentration response curve obtained with beclomethasone was compared to the curve of salbutamol, it was observed that beclomethasone inhibited the effects of insulin but less than that of salbutamol.

Since insulin is a pro-inflammatory and procontractile hormone (5), the potential protective effects of beclomethasone against insulin induced tracheal muscle contraction is presumably through its anti-inflammatory effects and its ability to prevent the release of prostaglandins and histamine which in turn inhibit airway hyper-responsiveness mediated by insulin (9). In another study it was observed that prolong exposure to insulin induced a hypercontractile phenotype leading to increased air-way reactivity due to its mitogenic potential, in isolated bovine tracheal muscle. This increased airway hyper-responsiveness was significantly inhibited in the presence of beclomethasone due to its ability to inhibit the proliferation of bovine tracheal muscle (17). So the author suggested that beclomethasone due to its antimitogenic effect can provide long term protection for those diabetic patients who regularly use inhalational insulin (17).

This *in-vitro *study provides the first evidence that salbutamol and beclomethasone can significantly inhibit the contractile response of insulin on guinea pig airways and salbutamol is more efficacious than beclomethasone in this regard. Insulin induced isolated tracheal muscle contraction in guinea pig model described in the present study closely resembles the bronchoconstriction induced by pulmonary delivery of inhaled insulin as airway smooth muscles are directly exposed to high concentration of insulin in both cases (4). So pretreatment with salbutamol may be preferred over beclomethasone for counteracting the respiratory adverse effects with inhaled insulin therapy.

## Conclusion

 Insulin has acute contractile effect on guinea pigs airway smooth muscle. Salbutamol is more efficacious than beclomethasone in amelioration of insulin induced tracheal tissue contraction. So we suggest that diabetic patients taking inhalational insulin may be pretreated with inhaled salbutamol rather than inhaled beclomethasone to ameliorate its potential respiratory adverse effects. 
